# Associations between Serum Apelin-12 Levels and Obesity-Related Markers in Chinese Children

**DOI:** 10.1371/journal.pone.0086577

**Published:** 2014-01-27

**Authors:** Hong-Jun Ba, Hong-Shan Chen, Zhe Su, Min-Lian Du, Qiu-Li Chen, Yan-Hong Li, Hua-Mei Ma

**Affiliations:** Pediatric Department, The First Affiliated Hospital, Sun Yat-sen University, Guangzhou, Guangdong Province, China; National Institute of Nutrition, India

## Abstract

**Objective:**

To investigate possible correlations between apelin-12 levels and obesity in children in China and associations between apelin-12 and obesity-related markers, including lipids, insulin sensitivity and insulin resistance index (HOMA-IR).

**Methods:**

Forty-eight obese and forty non-obese age- and gender-matched Chinese children were enrolled between June 2008 and June 2009. Mean age was 10.42±2.03 and 10.86±2.23 years in obesity and control groups, respectively. Main outcome measures were apelin-12, BMI, lipids, glucose and insulin. HOMA-IR was calculated for all subjects.

**Results:**

All obesity group subjects had significantly higher total cholesterol (TC), triglycerides (TG), low-density lipoprotein cholesterol (LDL-C), insulin levels and HOMA-IR (all P<0.05). In separate analyses, obese girls had significantly higher LDL-C, insulin and HOMA-IR than controls, and obese boys had significantly higher TC, TG, insulin and HOMA-IR than controls (all P<0.05). Apelin-12 levels were significantly higher in obese girls compared to controls (P = 0.024), and correlated positively with TG in all obese subjects. Among obese girls, apelin-12 levels correlated positively with TG, insulin and HOMA-IR after adjusting for age and BMI. In all boys (obese and controls) apelin-12 was positively associated with fasting plasma glucose (FPG). No significant correlations were found in either group between apelin-12 levels and other characteristics after adjusting for age, sex, and BMI.

**Conclusions:**

Apelin-12 levels are significantly higher in obese vs. non-obese girls in China and correlate significantly with obesity-related markers insulin, HOMA-IR, and TG. Increased apelin-12 levels may be involved in the pathological mechanism of childhood obesity.

## Introduction

The rapidly increasing prevalence of childhood obesity has alarmed public health agencies, healthcare clinicians and researchers, and the general public globally [1]. In China, the prevalence of obesity and overweight among children was 2% and 5%, respectively, in the 1980s; in 2002, 155 million children worldwide were overweight or obese, including 12 million in China [2]. Obesity is a multifactorial disease and its development is attributed to genetic predisposition, misregulation of energy balance, and environmental and social factors [3]. Obesity in childhood is associated with a variety of metabolic disorders, including insulin resistance [4], dyslipidemia [5], hyperglycemia [6], type 2 diabetes mellitus [7], and risk of cardiovascular complications [8]. Furthermore, obese children tend to become obese adults [9]. Clearly, understanding childhood obesity is critical in order to reduce its incidence and the development of related metabolic disorders.

Adipose tissue stores triglycerides and also secretes polypeptides, adipocyte-produced hormones called adipokines (or adipocytokines), including leptin, visfatin, vaspin, apelin, adiponectin and resistin, which all play important roles in metabolism and energy homeostasis [10]–[12]. Apelin, identified by Tatemoto et al. [12], is a novel bioactive peptide expressed in adipocytes of humans; it is encoded by the APLN gene and is the endogenous ligand of the orphan G protein-coupled receptor, APJ, now known as apelin receptor, APLNR. The gene encodes a 77-amino acid polypeptide [12], and all isoforms are derived from a common 77-amino acid precursor, preproapelin [13]. To date, 46 fragments of active apelin peptides have been identified from apelin-55 to apelin-12, all generated by the proproteins of 55 amino acids [14]. Active forms of apelin are expressed in many peripheral tissues (heart, lung, kidney, liver, adipose tissue, gastrointestinal tract, and endothelium) and brain regions (hypothalamus) [15]. Apelin-12 is a 12-amino peptide fragment that has been implicated in reducing blood pressure via a nitric oxide mechanism [16], and is involved in feeding mechanisms via stimulation of cholecystokinin secretion [17]. The synthesis of apelin in adipocytes is triggered by insulin and its plasma levels are reported to increase in association with insulin resistance and hyperinsulinemia [18]. Apelin-12 is considered to be one of the more potent forms of apelin [14].

Apelin expression participates in regulation of blood pressure [16], cardiac contractility [19], fluid balance [20] and stimulation of ACTH release by the pituitary [21]. Expression of apelin in adipocytes is shown to be increased in mouse models of obesity associated with hyperinsulinemia, and apelin levels paralleled plasma insulin levels during fasting and refeeding of mice [17]. Tumor necrosis factor-alpha (TNF-α) [22], growth hormone [23], insulin and glucocorticoids [24] all up-regulate the expression of apelin in adipocytes. Tasci et al. [25] have also found that plasma apelin-12 was lower in patients with elevated LDL-C. While Erdem et al. [26] found significantly reduced plasma apelin levels in obese subjects with untreated type 2 diabetes compared to non-diabetic subjects, Li et al. [27] have found elevated plasma apelin levels in people with impaired glucose tolerance and type 2 diabetes mellitus.

All studies cited above indicate that controversy exists around the levels and the associations of apelin in metabolic disorders. Although associations have been shown between apelin-12 and atopic dermatitis [28] and insulin resistance in adolescents with polycystic ovary syndrome [29], studies are lacking about associations of this adipocyte-secreted factor in obese children, and there is no agreement to date on whether apelin levels correlate with childhood obesity. Therefore, the purpose of the present study was to investigate possible correlations between apelin-12 levels and obesity in children in China and identify associations between apelin-12 and obesity-related markers, including lipids, insulin sensitivity and insulin resistance index.

## Patients and Methods

### Study Population and Anthropometric Measurements

Between June 2008 and June 2009, 88 (48 obese and 40 non-obese) Chinese children, matched for age and sex, were enrolled in this cross-sectional study. All participants were from Guangzhou, China, and recruited from patients receiving regular health check-ups or evaluation of obesity. Their demographic and clinical data were analyzed retrospectively. Using criteria developed in China, obesity was defined as BMI for age- and sex-specific categories at the 95th percentile or higher [30]. Exclusion criteria were the presence of endocrine diseases, infections, chronic illnesses, and use of prescription medication for any reason. BMI was calculated for all subjects (weight in kilograms divided by the square of height in meters). Waist circumference (WC) was measured with a tape measure at the level of the umbilicus to the nearest 0.1 cm as previously described [31]. Detailed personal and family medical history was obtained for each participant. All subjects underwent a complete physical examination. The stage of puberty was determined according to the Tanner criteria [32]. The nature and purpose of the study were carefully explained to both parents and subjects before obtaining written informed consent from parents and voluntary assent from the children. This study was approved by the Institutional Ethics Board of the First Affiliated Hospital of Sun Yat-sen University.

### Clinical Laboratory Determinations

Venous blood samples were drawn from all subjects in the morning between 8:00 AM and 9:00 AM after a 12-hour overnight fast. Blood samples were collected according to recommendations of the individual assay kits’ manufacturers. Serum and plasma samples were frozen and stored at −70°C until the tests were performed.

Laboratory determinations of plasma glucose (FPG, mg/dl), total cholesterol (TC, mmol/l) high-density lipoprotein cholesterol (HDL, mmol/l) and triglycerides (TG, mmol/l) were assessed by standard laboratory methods using commercially available test kits (Roche Diagnotics GmbH, Mannheim, Germany). Low-density lipoprotein cholesterol (LDL-C, mmol/l) value was obtained using the Friedwald formula. Other assays were performed as follows:

#### Insulin assay protocol

Insulin (µU/ml) was measured by an enzyme-linked immunoassay kit (DRG Instruments GmbH, Marburg, Germany), with a lower limit of sensitivity of 1.76 µU/ml and intra- and inter-assay CVs of 2.2% and 4.4%, respectively. Protocol was as follows: Add 25 µl/well of standard, sample, and positive control followed by 25 µl/well conjugate reagent and mix well for 10 sec. Incubate at room temperature (20–23°C) for 0.5 hours. Wash immunoplate 3 times with 400 µl/well of 1×assay buffer. Add 50 µl/well of enzyme complex and incubate at room temperature for 0.5 hours. Wash immunoplate 3 times with 400 µl/well of 1×assay buffer. Add 50 µl/well of TMB substrate solution and incubate at room temperature for 15 minutes. Terminate reaction with 50 µl/well of H2SO4. Read absorbance OD at 450 nm and calculate results.

#### Apelin-12 assay protocol

Serum apelin-12 levels were determined by enzyme-linked immunosorbent assay (ELISA) (Human Apelin-12 ELISA Kit. Phoenix Pharmaceuticals, Belmont, CA) Intra- and inter-assay CVs were 5%, and 14%, respectively. Protocol was as follows: Add 50 µl/well of standard, sample, or positive control, 25 µl primary antibody and 25 µl biotinylated peptide. Incubate at room temperature (20–23°C) for 2 hours. Wash immunoplate 4 times with 350 µl/well of 1×assay buffer. Add 100 µl/well of SA-HRP solution and incubate at room temperature for 1 hour. Wash immunoplate 4 times with 350 µl/well of 1×assay buffer. Add 100 µl/well of TMB substrate solution and incubate at room temperature for 1 hour. Terminate reaction with 100 µl/well of 2N HCI. Read absorbance OD at 450 nm and calculate results.

#### Estradiol & testosterone assays

Serum hormone levels (estradiol and testosterone) were measured by chemiluminescence microparticle immunoassay kits (CMIA) (ARCHITECT Integrated System, Abbott Laboratories, Lake Forest, IL, USA), with lower limits of sensitivity of 10 pg/ml for estradiol and 0.14 ng/mL for testosterone. Intra- and inter-assay CVs were 4.5% and 6.1% for estradiol, and 2.6% and 4.9% for testosterone, respectively. Protocols for both assays were performed as follows: Load reagents (estradiol/testosteron) onto the ARCHITECT *i* System. Mix calibrators and controls by gentle inversion prior to use. Hold bottles vertically and dispense 15 drops (400 µl) of each calibrator or 10 drops (250 µl) of each control into each respective sample cup. Load samples and press RUN. The ARCHITECT *i* System performs all mixing and reaction functions, incubation, washing, measurements and calculations.

#### Insulin resistance index (HOMA-IR)

The index of insulin resistance, HOMA-IR, was calculated according to the following homeostasis model assessment (HOMA), as previously described [33]: HOMA-IR =  FINS×FPG/22.5, where FINS is fasting insulin (*µ*U/mL), and FPG is fasting plasma glucose level (mmol/l).

### Statistical Analysis

Subjects’ demographics and clinical characteristics were summarized as mean with standard deviation (mean±SD) for continuous data and n(%) for categorical data by group; differences between groups were compared using two-sample t-test for continuous data, Pearson Chi-square test for sex and Mann-Whitney U test for non-normally distributed data (Tanner criteria for stage of puberty). Correlations of apelin-12 levels with demographics and clinical characteristics were presented as coefficients of correlation (r) with respective *P*-values through partial correlation analysis after adjusting for age, sex, and BMI overall, and by adjusting for age and BMI in boys and girls separately. General linear regression analysis was performed to identify associations of apelin-12 levels with obesity-related markers by adjusting for age, sex, and BMI in groups overall, and by adjusting for age and BMI in boys and girls separately. Results were presented as estimated β with standard error (SE) and P-value. All statistical assessments were two-tailed and P<0.05 was set as statistical significance. All statistical analyses were performed using SPSS 17.0 statistics software (SPSS Inc, Chicago, IL, USA).

## Results

A total of 88 subjects were enrolled in this study, including 48 subjects (20 girls/28 boys) in an obesity group and 40 subjects (16 girls/24 boys) in a non-obese control group. Average ages were 10.42 (SD = 2.03) years and 10.86 (SD = 2.23) years in the obesity group and control group, respectively. Table 1 summarizes subjects’ demographics and clinical characteristics by groups overall and in males and females separately. Among all subjects, those in the obesity group had significantly higher TC, TG, LDL-C, insulin, and HOMA-IR (all P<0.05) than the control group. When males and females were analyzed separately, obese girls had significantly higher LDL-C, insulin, HOMA-IR, and apelin-12 than controls; obese boys had significantly higher TC, TG, insulin, and HOMA-IR than controls. No significant differences were found in apelin-12 levels between obese and non-obese groups. (Table 1).

**Table 1:** Demographics and clinical characteristics of all subjects by obese and control groups and boys and girls separately.

	Overall			Girls			Boys		
Variables	Control (n = 40)	Obesity (n = 48)	*P*-value	Control (n = 16)	Obesity (n = 20)	*P*-value	Control (n = 24)	Obesity (n = 28)	*P*-value
Age, years	10.86±2.23	10.42±2.03	0.334	10.85±2.23	9.73±2.08	0.129	10.87±2.28	10.91±1.88	0.941
Sex			0.874						
Girls	16 (40%)	20 (41.7%)							
Boys	24 (60%)	28 (58.3%)							
BMI, Kg/m2	16.80±1.83	25.65±4.18	<.001*	16.76±2.21	24.02±3.90	<.001*	16.83±1.58	26.86±4.04	<.001*
Tanner stage			0.085			0.223			0.351
1	23 (57.5%)	18 (37.5%)		9 (56.3%)	6 (60%)		14 (58.3%)	12 (42.9%)	
2	7 (17.5%)	11 (22.9%)		1 (6.3%)	0 (0%)		6 (25%)	11 (39.3%)	
3	4 (10%)	11 (22.9%)		1 (6.3%)	8 (40%)		3 (12.5%)	3 (10.7%)	
4	4 (10%)	3 (6.3%)		3 (18.8%)	1 (5%)		1 (4.2%)	2 (7.1%)	
5	2 (5%)	5 (10.4%)		2 (12.5%)	5 (25%)		0 (0%)	0 (0%)	
TC, mmol/L	4.08±0.76	4.69±0.64	0.003*	4.37±0.36	4.77±0.74	0.097	3.92±0.88	4.63±0.55	0.007*
TG mmol/L	0.89±0.30	1.27±0.62	0.003*	1.14±0.32	1.30±0.75	0.613	0.75±0.20	1.25±0.51	<.001*
LDL-C, mmol/L	2.38±0.75	2.90±0.73	0.037*	2.34±0.55	3.11±0.71	0.041*	2.40±0.91	2.75±0.72	0.308
HDL-C mmol/L	1.56±0.23	1.46±0.57	0.579	1.58±0.25	1.39±0.24	0.149	1.54±0.23	1.52±0.73	0.930
Insulin, µU/ML	7.62±4.93	23.63±17.57	<.001*	7.48±4.03	16.09±7.63	<.001*	7.71±5.53	29.01±20.59	<.001*
FPG, mmol/L	4.86±0.52	4.80±0.54	0.593	4.65±0.36	4.58±0.62	0.683	5.01±0.57	4.96±0.42	0.745
HOMA-IR	1.72±1.10	4.73±3.17	<.001*	1.42±0.51	3.21±1.78	<.001*	1.91±1.33	5.80±3.50	<.001*
Apelin-12 level, ng/Ml	1.13±0.55	1.20±0.59	0.576	0.84±0.28	1.19±0.57	0.024*	1.31±0.60	1.20±0.61	0.537
Serum estradiol level	32.04±41.06	24.80±24.26	0.437	50.00±54.40	32.33±32.07	0.328	15.46±6.24	17.27±8.04	0.509
Serum testosterone level	1.08±1.62	0.65±0.69	0.181	0.47±0.77	0.53±0.46	0.762	1.51±1.92	0.72±0.80	0.123

BMI, body mass index; FPG, fasting plasma glucose; HOMA-IR, homeostasis model assessment of insulin resistance; HDL-C, high density lipoprotein cholesterol; LDL-C, low density lipoprotein cholesterol; TC, total cholesterol; TG, triglycerides.

Continuous data are summarized as mean±SD and categorical data as n (%); differences between the groups were compared using two-sample t-test for continuous data, Pearson Chi-square test for sex, and Mann-Whitney U test for Tanner stage.

* indicates significant difference between groups. (*P*<0.05).

**Figure 1:**
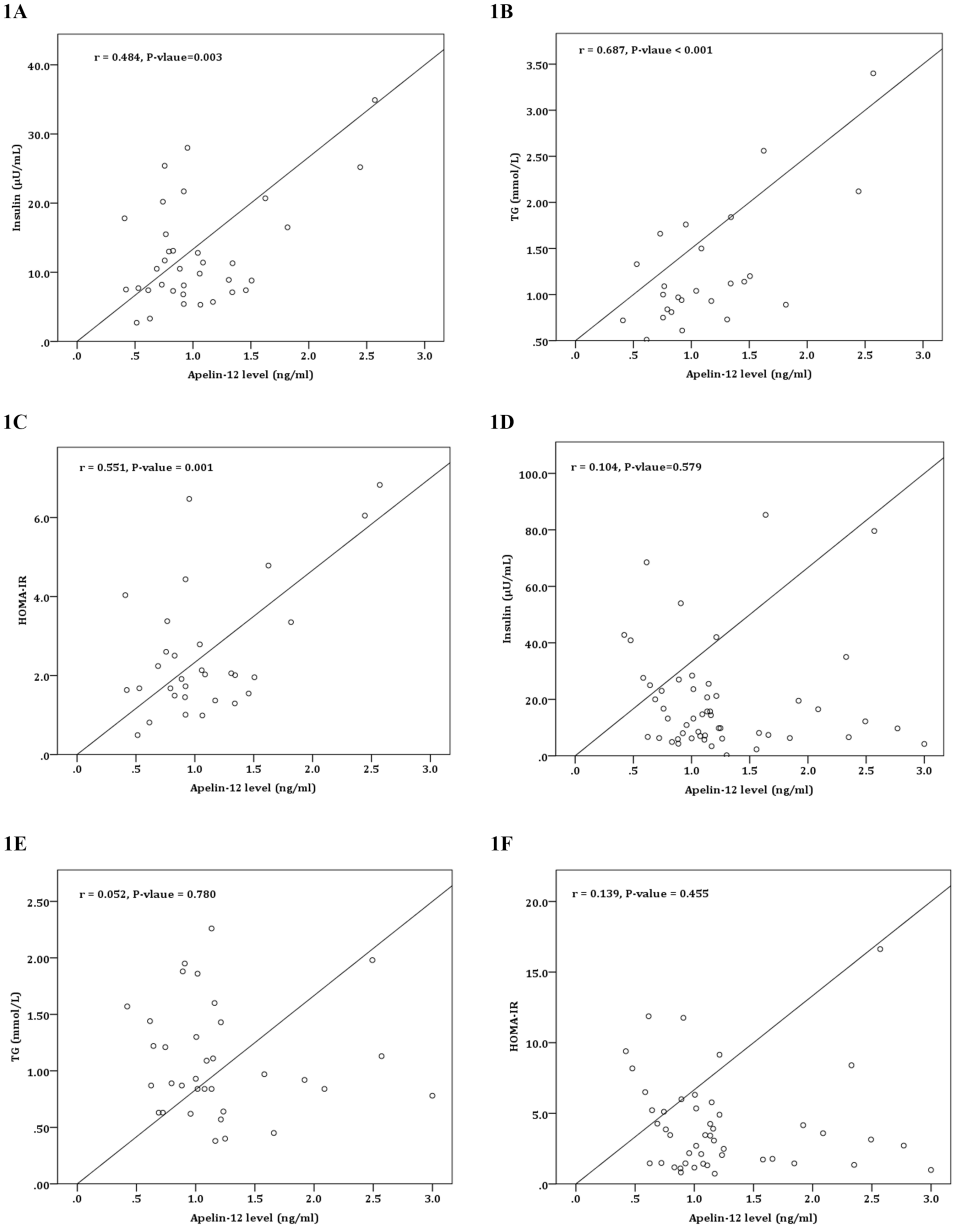
Associations between apelin levels, fasting insulin, triglycerides and HOMA-IR. Figure 1 depicts the association between apelin levels and (A) fasting insulin, (B) TG, and (C) HOMA-IR in female subjects and the association between apelin levels and (D) fasting insulin, (E) TG, and (F) HOMA-IR in male subjects. Correlation coefficients with respective P-values are presented through partial correlation analysis after adjusting for age and BMI. The apelin levels were positively correlated with fasting insulin, TG, and HOMA-IR in females; No significant results were observed in males. TG, triglycerides; HOMA_IR, homeostasis model assessment of insulin resistance; BMI, body mass index.

Apelin-12 levels correlated positively with insulin (A), TG (B) and HOMA-IR (C) in girls, however apelin-12 levels were not correlated significantly with insulin, TG and HOMA-IR in boys (D–F) (Fig. 1). Table 2 represents partial correlation analysis of apelin-12 levels with other characteristics after adjusting for age, sex, and BMI in all subjects, and girls and boys in the obese and control groups separately, adjusting for age and BMI. In the obesity group overall, apelin-12 correlated positively with TG but no other significant correlations were found in either of the groups between apelin-12 levels and other characteristics after adjusting for age, sex, and BMI. Among females in the obese group, apelin-12 levels correlated positively with TG, insulin, and HOMA-IR after adjusting for age and BMI.

**Table 2:** Correlation of apelin-12 levels with demographics and clinical characteristics by obesity and control groups, and boys and girls separately.

	Overall		Girls		Boys	
	r	*P*-value	r	*P*-value	r	*P*-value
Obesity group						
TC, mmol/L	0.162	0.337	0.121	0.642	0.194	0.427
TG mmol/L	0.436	0.006*	0.723	0.001*	0.083	0.728
LDL-C, mmol/L	0.165	0.351	−0.063	0.830	0.299	0.214
HDL-C mmol/L	−0.190	0.289	−0.387	0.172	−0.130	0.607
Insulin, µU/ML	0.186	0.226	0.600	0.008*	0.152	0.467
FPG, mmol/L	0.207	0.183	0.017	0.947	0.207	0.333
HOMA-IR	0.217	0.168	0.580	0.015*	0.130	0.546
Serum estradiol level	0.289	0.121	0.384	0.158	0.384	0.175
Serum testosterone level	−0.030	0.852	−0.386	0.140	−0.047	0.827
Control group						
TC, mmol/L	0.48	0.082	0.882	0.118	0.592	0.093
TG mmol/L	−0.058	0.843	0.833	0.167	−0.045	0.908
LDL-C, mmol/L	0.525	0.147	0.913	0.268	0.709	0.180
HDL-C mmol/L	0.184	0.635	−0.765	0.445	0.239	0.699
Insulin, µU/ML	−0.112	0.514	0.129	0.675	−0.130	0.565
FPG, mmol/L	0.385	0.037*	0.246	0.467	0.448	0.062
HOMA-IR	−0.066	0.733	0.094	0.796	0.13	0.546
Serum estradiol level	−0.152	0.500	−0.331	0.350	0.275	0.412
Serum testosterone level	0.075	0.718	0.393	0.261	0.034	0.904

BMI, body mass index; FPG, fasting plasma glucose; HOMA-IR, homeostasis model assessment of insulin resistance; HDL-C, high density lipoprotein cholesterol; LDL-C, low density lipoprotein cholesterol; TC, total cholesterol; TG, triglycerides.

Results are presented as coefficients of correlation (r) with respective *P*-value through partial correlation analysis after adjusting for age, sex, and BMI overall, and after adjusting for age and BMI in boys and girls separately.

* indicates significant correlation. (*P*<0.05).

Table 3 shows results of general linear regression analysis of the association of apelin 12 levels with group effect, presenting subjects’ characteristics after adjusting for age, sex and BMI. Apelin-12 levels increased as TG increased in all subjects and in girls alone [β(SE) = 0.336 (0.142) in all subjects, P = 0.021; β(SE) = 0.074(0.263) in boys, P = 0.780; β(SE) = 0.499 (0.129), P = 0.001 in girls]. When the data were segregated by gender, apelin-12 in obese girls was positively associated with TG, insulin, and HOMA-IR. [TG: β(SE) = 0.579 (0.143), P = 0.002; insulin: β(SE) = 0.056 (0.019), P = 0.008; β(SE) = 0.243 (0.088), P = 0.015]. In all male subjects (control and obesity groups), apelin-12 was positively associated with FPG. [β(SE) = 0.448(0.191), P = 0.024] (Table 3).

**Table 3:** Linear regression analysis of associations of apelin-12 levels with obesity-related markers.

	Overall (Adjusted for age, sex, BMI)		Control group (Adjusted for age, sex, BMI)		Obesity group (Adjusted for age, sex, BMI)	
Variables	β(SE)	*P*-value	β(SE)	*P*-value	β(SE)	*P*-value
Total (N = 88)						
Group						
Obesity	0.208 (0.224)	0.356	–		–	
Control	1					
Tanner stage	0.070 (0.073)	0.343	0.057 (0.115)	0.614	0.012 (0.112)	0.918
TC, mmol/L	0.178 (0.112)	0.118	0.399 (0.210)	0.082	0.146 (0.150)	0.973
TG mmol/L	0.336 (0.142)	0.021*	−0.161 (0.793)	0.843	0.415 (0.143)	0.006*
LDL-C, mmol/L	0.191 (0.112)	0.096	0.429 (0.263)	0.147	0.129 (0.136)	0.351
HDL-C mmol/L	−0.173 (0.169)	0.313	0.480 (0.967)	0.497	−0.186 (0.172)	0.289
Insulin, µU/ML	0.005 (0.005)	0.373	−0.012 (0.018)	0.514	0.007 (0.006)	0.226
FPG, mmol/L	0.303 (0.131)	0.024*	0.442 (0.193)	0.037*	0.245 (0.181)	0.183
HOMA-IR	0.035 (0.031)	0.267	−0.034 (0.100)	0.733	0.049 (0.035)	0.168
Serum estradiol level	0.002 (0.003)	0.382	−0.002 (0.002)	0.500	0.009 (0.005)	0.121
Serum testosterone level	0.041 (0.068)	0.547	0.030 (0.083)	0.718	−0.031 (0.163)	0.852
Boys (N = 52)						
Group						
Obesity	0.044 (0.336)	0.895				
Control	1					
Tanner stage	0.166 (0.140)	0.242	0.190 (0.224)	0.407	0.161 (0.200)	0.430
Total cholesterol, mmol/L	0.250 (0.154)	0.115	0.490 (0.252)	0.093	0.216 (0.265)	0.427
TG, mmol/L	0.074 (0.263)	0.780	−0.163 (1.354)	0.908	0.096 (0.273)	0.728
LDL-C, mmol/L	0.258 (0.148)	0.093	0.588 (0.337)	0.180	0.242 (0.187)	0.214
HDL-C mmol/L	−0.062 (0.196)	0.754	0.774 (1.821)	0.699	0.103 (0.196)	0.607
Insulin, µU/ML	0.003 (0.006)	0.587	−0.016 (0.027)	0.565	0.005 (0.006)	0.467
FPG, mmol/L	0.448 (0.191)	0.024*	0.533 (0.266)	0.062	0.301 (0.304)	0.333
HOMA-IR	0.017 (0.036)	0.644	−0.039 (0.130)	0.767	0.024 (0.039)	0.546
Serum estradiol level	0.038 (0.019)	0.056	0.027 (0.032)	0.412	0.041 (0.028)	0.175
Serum testosterone level	0.001 (0.087)	0.991	0.016 (0.131)	0.904	−0.043 (0.193)	0.827
Girls (N = 36)						
Group						
Obesity	0.182 (0.301)	0.549				
Control						
Tanner stage	−0.010 (0.088)	0.909	−0.049 (0.101)	0.638	−0.022 (0.131)	0.871
TC, mmol/L	0.099 (0.163)	0.548	0.354 (0.134)	0.118	0.091 (0.192)	0.642
TG, mmol/L	0.499 (0.129)	0.001*	0.465 (0.218)	0.167	0.579 (0.143)	0.002*
LDL-C, mmol/L	−0.022 (0.171)	0.898	0.312 (0.140)	0.268	−0.047 (0.214)	0.830
HDL-C mmol/L	−0.635 (0.522)	0.240	−0.659 (0.554)	0.445	−1.089 (0.750)	0.172
Insulin, µU/ML	0.035 (0.015)	0.027*	0.009 (0.022)	0.675	0.056 (0.019)	0.008*
FPG, mmol/L	0.076 (0.167)	0.654	0.208 (0.274)	0.467	0.018 (0.257)	0.947
HOMA-IR	0.210 (0.074)	0.009*	0.056 (0.210)	0.796	0.243 (0.088)	0.015*
Serum estradiol level	0.002 (0.003)	0.352	−0.002 (0.002)	0.350	0.007 (0.005)	0.158
Serum testosterone level	−0.056 (0.167)	0.743	0.140 (0.116)	0.261	−0.579 (0.370)	0.140

BMI, body mass index; FPG, fasting plasma glucose; HOMA-IR, homeostasis model assessment of insulin resistance; HDL-C, high density lipoprotein cholesterol; LDL-C, low density lipoprotein cholesterol; TC, total cholesterol; TG, triglycerides.

Results are presented as estimated β with standard error (SE) and *P*-value through general linear regression analysis after adjusting for age, sex, and BMI overall, and after adjusting for age and BMI in boys and girls separately.

* indicates significant association. (*P*<0.05).

### Correlation Analysis of Apelin in Obese and Nonobese Subjects

Correlation analysis of apelin-12 levels with anthropometric and biochemical indices, demonstrated significant correlations between apelin-12 and BMI-SDS, TG, FINS, and HOMA-IR in all girl subjects (obese and non-obese groups). No correlations were found between apelin-12 levels and estradiol and testosterone; no significant differences were found in estradiol and testosterone levels between girls and boys in both groups. In boys, FPG levels correlated positively with apelin-12 levels (Table 2). Stepwise linear regression analysis with apelin-12 as a dependent variable and BMI-SDS, TG, FINS, and HOMA-IR as independent variables revealed that only TG was an important determinant of apelin-12 levels in female subjects. (Table 3).

## Discussion

The present study analyzed the data of obese and non-obese children in Guangzhou, China, aiming to determine whether correlations could be found between apelin-12 levels of the groups and obesity in this population and to identify associations, if any, between apelin-12 and obesity-related markers, especially lipids, insulin sensitivity and insulin resistance index. In obese subjects, TC, TG, LDL-C, insulin, and HOMA-IR were significantly higher than in non-obese controls. When boys and girls were analyzed separately, altogether different results were shown: obese girls had significantly higher LDL-C, insulin, and HOMA-IR than controls; on the other hand, obese boys had significantly higher TC, TG, insulin, and HOMA-IR than controls. Notably, apelin-12 levels were significantly higher in obese girls compared to controls (p = 0.024). Age-, sex- and BMI-adjusted correlation analysis of apelin-12 levels with all other obesity-related factors revealed a positive correlation only between apelin-12 and TG, but no other significant correlations were found in either group between apelin-12 levels and the other characteristics. Most importantly, apelin-12 levels correlated positively with TG, insulin, and HOMA-IR in obese girls, indicating clinical significance although the mechanism remains to be explained. Also, linear regression analysis showed that apelin-12 levels increased as TG increased in all subjects and in girls alone, showing again that apelin-12 was positively associated with TG, insulin, and HOMA-IR. Among all boys in the control and obese groups, apelin-12 was positively associated with FPG only.

To the best of our knowledge, this is the first study on apelin-12 and obesity-related markers in children in China. Our results showed significantly higher TC, TG, LDL-C, insulin, and HOMA-IR in all obese subjects versus non-obese controls, but results vary among other international studies of apelin and obesity-related markers. Boucher et al. [17] reported increased plasma apelin levels in obese adult males and suggested further that, since adipose tissue is an important source of apelin production, and the expression of apelin and APJ both increase in adipose tissue in obese individuals, elevated serum apelin of obese subjects might be attributable to augmented adipose tissue. Minor changes in plasma apelin were associated with changes in BMI in obese subjects during 8-weeks of a very-low-calorie diet [34]. Those authors have suggested that apelin may not correlate as strongly with fat mass as with more abundant adipokines such as leptin and adiponectin. Li et al. [27] have found a correlation between apelin and BMI, and Heinonen et al. [35] have also found that apelin plasma concentration was significantly higher in morbidly obese patients compared to normal-weight controls. In contrast, Reinehr and colleagues (2011) evaluated apelin concentration, weight status, body fat, insulin resistance, leptin and obesity-related cardiovascular risk factors before and after one-year lifestyle intervention, demonstrating that weight loss in obese children was not associated with changes in apelin concentration as the authors have hypothesized, and no significant relationships were found between apelin, insulin resistance, cardiovascular risk factors and obesity in children [36]. Results of these studies indicate that agreement is still lacking on the relationship between apelin levels and obesity, especially in children.

Gender differences in relation to apelin-12 levels have not been fully explored or explained. Significantly lower apelin levels reported in pubertal obese children compared to normal-weight pubertal children suggests a link between sex hormones and apelin levels [37]. A study focused on gender differences of pubertal adiponectin levels in association with serum androgen levels found significantly reduced serum adiponectin concentration in boys, which was in parallel to physical and pubertal development compared to those adiponectin concentrations of age-matched female counterparts [38]. The decline was inversely related to testosterone and dehydroepiandrosterone sulfate levels, and the authors reported a strong association between adiponectin levels and obesity, pubertal development and metabolic parameters. The present study found significantly different results between boys and girls of the obese group: in this group, girls had significantly higher LDL-C, insulin, and HOMA-IR vs. controls while obese boys had significantly higher TC, TG, insulin and HOMA-IR vs. controls. Most importantly, apelin-12 levels were significantly higher in all obese female children vs. non-obese controls, which was not true for boys. Gender differences in our study require further investigations, however evidence pertaining to adioponectin and pubertal development sheds light on the differences between male and female apelin-12 levels and and its association with other biochemical parameters.

Correlations between apelin and insulin resistance, a major characteristic of obesity and type 2 diabetes, have been demonstrated by several authors. Erdem et al. [26] demonstrated a negative correlation with HOMA-IR in newly diagnosed type 2 diabetes mellitus. Tasci et al. [25] have reported a mild to moderate negative correlation between apelin and HOMA-IR in patients with elevated LDL-C. In contrast, Li et al. [27] described a positive correlation with HOMA-IR in patients with impaired glucose tolerance and type 2 diabetic subjects, and Hosoya et al. [18] have shown that plasma apelin levels increased markedly in insulin resistance and hyperinsulinemia. In the present study, HOMA-IR was significantly higher in all obese subjects compared to that in non-obese controls. Taken together, our results and those reported previously indicate that different associations between apelin and insulin resistance may depend on the extent of insulin resistance. Insulin resistance is common to both obesity and type 2 diabetes, and apelin is linked with obesity-associated variations of insulin sensitivity status [17]. In view of these associations, Castan-Laurell et al. [15] suggest that apelin may act as an insulin sensitizing agent and may be a potential target for diabetes treatment, that is, given its potent activity in energy metabolism and ability to improve insulin sensitivity.

In the present study, positive correlations were found between apelin-12 levels and TG, insulin, and HOMA-IR in obese girls. TG, as one of the components of metabolic syndrome, plays an important role in predicting impaired glucose tolerance in adolescents at risk for type 2 diabetes mellitus [39], and it has been suggested as a surrogate to identify insulin resistance in apparently healthy subjects [40]. In the present study, although we found a strong correlation between TG and serum apelin concentration in obese girls after adjusting for BMI and age, the relationship was insignificant in non-obese subjects, suggesting that the association may be more pronounced above a certain threshold determined by the extent of obesity, or that TG may affect apelin levels by inducing insulin resistance. Than et al. [41] proposed that apelin inhibits both adipogenesis and lipolysis through specific molecular pathways, acting through apelin APJ receptors expressed in adipocytes and resulting in decreased levels of free fatty acids in plasma and the release of free fatty acids from adipocytes. Yue et al. showed that mice deficient in apelin signaling also have increased circulating levels of free fatty acids and glycerol along with increased adiposity, and these effects can be reversed by exogenous apelin [42]. Since high levels of free fatty acid can lead to insulin resistance, this may help to explain the beneficial role of apelin in regulating metabolic homeostasis, although further study is needed to describe the underlying mechanism.

This study has some limitations, including that the data were cross-sectional and analyzed retrospectively from a relatively small cohort. Also, we did not investigate the mechanisms underlying apelin-12 levels in association with weight status and obesity-related markers. Further prospective studies with large samples are needed to clarify the role and mechanisms of apelin in association with obesity-related markers in a sub-population of obese children.

In conclusion, apelin-12 levels are significantly higher in obese female children in China compared to non-obese and correlate significantly with the obesity-related markers insulin, HOMA-IR and TG in this population. Increased apelin-12 levels may be involved in the pathological mechanism of obesity. Our results suggest beneficial effects of apelin in maintaining metabolic homeostasis and its potential clinical utility as a biomarker or therapeutic target.
